# The Role of Superior Temporal Cortex in Auditory Timing

**DOI:** 10.1371/journal.pone.0002481

**Published:** 2008-06-25

**Authors:** Domenica Bueti, Eelco V. van Dongen, Vincent Walsh

**Affiliations:** 1 Institute of Cognitive Neuroscience, University College London, London, United Kingdom; 2 Department of Psychology, University College London, London, United Kingdom; University of Southern California, United States of America

## Abstract

Recently, there has been upsurge of interest in the neural mechanisms of time perception. A central question is whether the representation of time is distributed over brain regions as a function of stimulus modality, task and length of the duration used or whether it is centralized in a single specific and supramodal network. The answers seem to be converging on the former, and many areas not primarily considered as temporal processing areas remain to be investigated in the temporal domain. Here we asked whether the superior temporal gyrus, an auditory modality specific area, is involved in processing of auditory timing. Repetitive transcranial magnetic stimulation was applied over left and right superior temporal gyri while participants performed either a temporal or a frequency discrimination task of single tones. A significant decrease in performance accuracy was observed after stimulation of the right superior temporal gyrus, in addition to an increase in response uncertainty as measured by the Just Noticeable Difference. The results are specific to auditory temporal processing and performance on the frequency task was not affected. Our results further support the idea of distributed temporal processing and speak in favor of the existence of modality specific temporal regions in the human brain.

## Introduction

Temporal information is embedded in many aspects of our experience-the music we listen to and the traffic lights we see have meaning partly because of the temporal information they contain. Time, however, unlike other features of the sensory world like color or pitch, does not have a dedicated receptor system, yet temporal information is contained in visual, auditory and tactile sensory inputs. The question arises, therefore, of whether temporal processing relies on either modality specific or supramodal mechanisms. The former of these seem increasingly likely since our experience of time differs among modalities [1]–[4], and sensory areas are involved in temporal processing [5], [6]. Neuroimaging, neurophysiological, neuropsychological and Transcranial Magnetic Stimulation (TMS) studies investigating the neural correlates of temporal processing do not conclusively address this issue [7]–[9]. Apart from a more established role of the basal ganglia and the cerebellum as time generators [10]–[13], the role of many cortical areas, including sensory specific cortices, remain more controversial. Most of these studies are either focused on the role of associative cortices such as parietal and dorsolateral prefrontal areas, or interested in exploring the basic core of the time network. Despite this emphasis on either subcortical timing centers or higher cortical areas, some neuroimaging studies report activations of the superior temporal gyrus. These activations were associated with temporal discrimination and reproduction of auditory stimuli [14]–[16] as well as with the production of rhythms learned through the auditory modality [17]–[20]. Visual analogues of such activity have been reported in extrastriate visual areas during discrimination and reproduction of visual durations [21], [22], and in association with visually guided timed motor responses [20], [23].

Although it is often not commented upon, activity in modality specific areas during temporal tasks has been observed. As far as we know, only two functional Magnetic Resonance Imaging (fMRI) studies have observed activity in superior temporal cortex in the temporal discrimination of visual stimuli [24], [25]. But this activity, as suggested by the authors, may reflect the subjects’ strategy of using auditory imagery. It is well known indeed, that the discrimination of temporal intervals is more difficult for visual than for auditory stimuli [26]. Therefore is not implausible that participants use auditory imagery or subvocalization as help in the discrimination of visual durations. In a previous TMS study we transiently disrupted activity in extrastriate visual cortex (V5/MT) and in left and right inferior parietal cortex while subjects discriminated visual and auditory durations. We found that the right posterior parietal cortex was important for timing of auditory and visual stimuli and that MT/V5 was necessary only for timing of visual events [6]. The present study represents an extension of this work, and aims to investigate the role of the auditory cortex in auditory timing. We stimulated the left and the right superior temporal cortex at a site that Molholm and colleagues found evidence for detection of single tones of different duration and frequency. [27]. In order to dissociate the processing of auditory timing information from that of low level auditory features, we used pitch discrimination as a control task.

## Materials and Methods

### Subjects

We tested eight healthy subjects (seven males; mean age was 28.2 years). All participants gave written consent and were treated in accordance with the Declaration of Helsinki. They all had previous experience of repetitive Transcranial Magnetic Stimulation (rTMS) but were naïve to the purpose of the experiment at hand. The experimental protocol was approved by the University College London Research Ethics committee, protocol ID #1144/001.

### Stimuli and procedure

We used an auditory duration and an auditory frequency discrimination task. Participants were tested on each task in separate sessions, in two different days. The order of the sessions/tasks was counterbalanced across participants. The auditory stimuli were single tones presented through headphones (Sennheiser PXC 250) at 70 dB volume level. The headphone setup included both passive and active noise cancellation systems that dampened environmental noise by 15 dB to overcome the noise made by the TMS pulses. Using TMS on line with auditory stimuli potentially presents special problems because the auditory clicks it produces may interfere with the perception of the stimuli. The problem may be greater in duration experiments because auditory clicks can sometimes interfere with temporal perception. We arrived at our arrangement with headphones following pilot experiments and other experiments with temporal discriminations (Bueti et al., 2008). In addition to the sound attenuation we considered it important to block TMS trials so that subjects were never in a state of uncertainty about the timing of the TMS. We also controlled for any potential non specific effects by applying TMS over three control sites. Although no visual stimuli were presented during trials, a 19”colour monitor (800×600 pixels, 75 Hz refresh rate, 100 cm viewing distance) was used to present performance feedback to the participant during the practice block only. The screen was otherwise blank throughout the experiment.

In both tasks two tones varying either in duration or frequency were presented in sequence. In both tasks the first tone (600 ms duration and 1000 Hz frequency) was the standard tone, while the second was one of six comparison tones (three longer/higher and three shorter/lower than the standard). The difference between the standard and the comparisons was ±10, ±20 ±40 milliseconds in the duration task and ±1, ±2 ±4 Hz in the frequency task. The choice of the different duration and frequency steps was based on a pilot study. Each trial started with the presentation of the standard tone followed, after a brief blank interval (1000 ms) by the comparison tone. The intertrial interval was two seconds. Participants had to select one of two response keys to indicate which of the two tones was longer in duration (duration task) or higher in pitch (frequency task).

Each block consisted of the combination of the standard duration or standard frequency followed by one of six comparison durations/frequencies presented five times, for a total of 30 trials. The six comparison durations and frequencies were randomly ordered. Stimuli, responses and TMS triggering were generated and measured by E-Prime software running on an IBM compatible Pentium IV computer.

During both tasks, participants were seated in a massage chair in which they laid facedown in a headrest for added comfort and head stability.

### Transcranial Magnetic Stimulation

A Magstim Super Rapid Stimulator (Magstim, UK) was used to deliver TMS via a figure-of-eight coil with a diameter of 50 mm. Its maximal output was two Tesla. TMS was delivered at 65% of maximal stimulator output, with the coil handle pointing backwards and parallel to the midline. A single intensity was used for all subjects because it is known that motor threshold cannot be assumed to be a guide to sensory cortex excitability and it is therefore an inappropriate guide to the cortical excitability of other non-motor areas of the brain (Stewart, Walsh, & Rothwell, 2001). This level of intensity was suprathreshold for phosphene and motor thresholds for all subjects. TMS was delivered at the onset of the standard tone, and was a train of five pulses lasting 500 ms, 10 Hz in frequency.

Each experimental session started with a practice block of 60 trials without TMS.

The first 50% of the trials in the practice block was followed by a feedback on participants’ performance. Subjects were trained without TMS in order to get familiar with the task and to reach a stable level of performance. The order of the TMS sites was counterbalanced across subjects. The vertex was chosen as a control site to control for non specific effects of TMS such as acoustic and somatosensory artifacts. Because we chose the vertex stimulation as baseline condition, the trials without TMS were not included in any analysis.

Each experimental session, one for each task, consisted of 12 blocks of trials, four blocks for each TMS site. The total number of observations for each duration/frequency step at each TMS site was equal to 20.

### Anatomical localization

**Figure 1:**
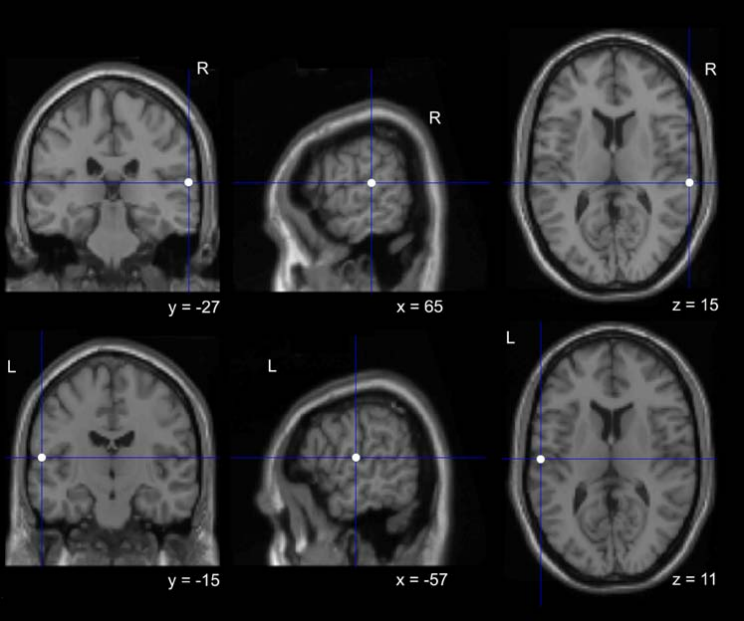
Brain sites of stimulation. MNI coordinates on the single subject MNI template used to localize the right and left STG.

**Figure 2:**
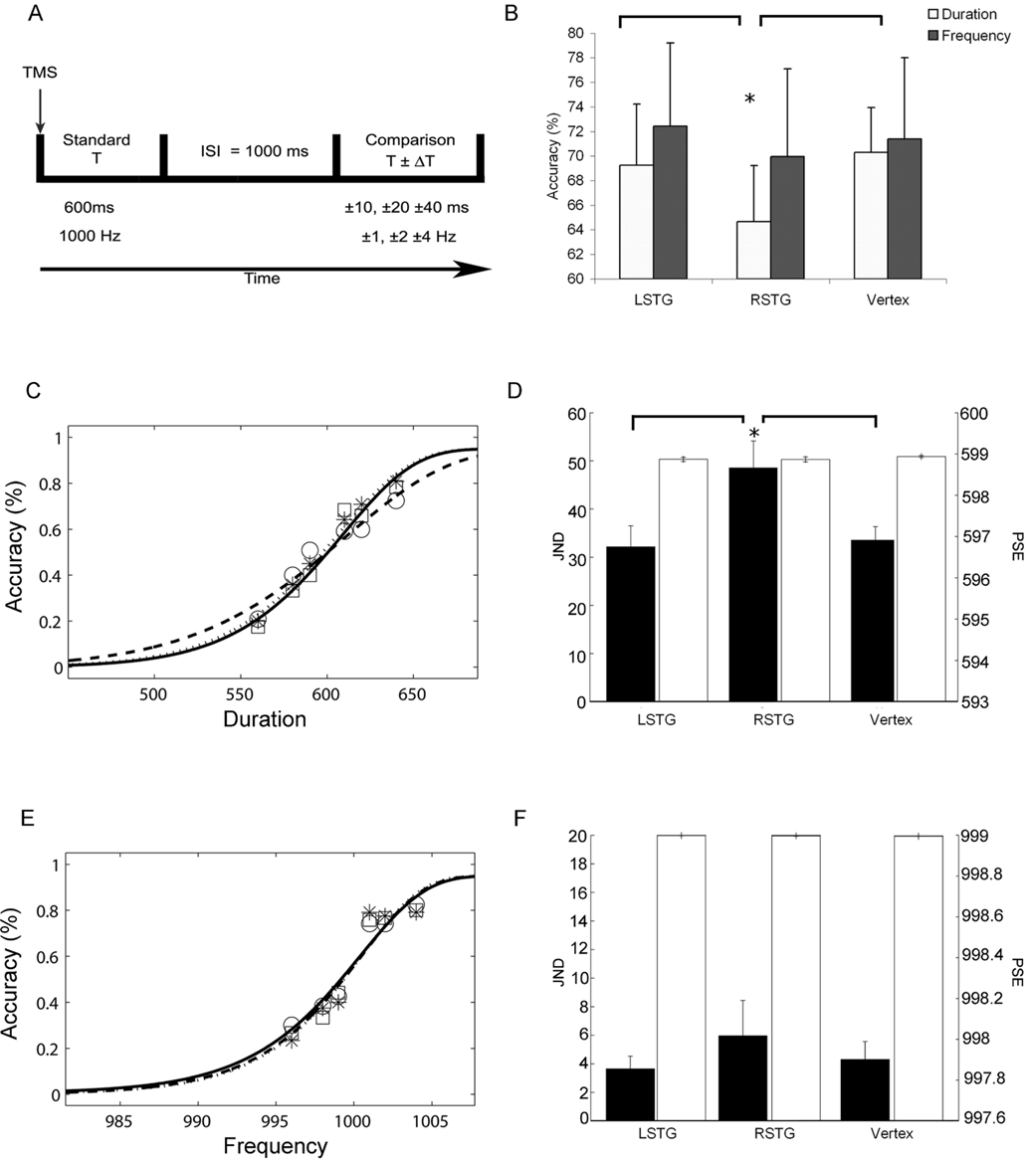
Results. A) Timeline of the experimental paradigm; B) Percentage of accurate responses and standard errors across the three TMS sites for the duration (white bars) and the frequency (black bars) discrimination tasks. Asterisk indicates significant differences in Tukey’s tests (* p<0.02). In order to check that the two tasks were well matched in terms of difficulty we also compared the two tasks after vertex stimulation (vertex Duration versus vertex Frequency: T_7_ = −0.21 P = 0.8). C–E) Group accuracy data (number of hits/total number of targets) across different comparison durations, for rTMS of the vertex (□), right (○) and left (*) STG. The line fitting the symbols are averaged fitted functions (obtained fitting the group averaged data). Solid lines represent rTMS of the vertex, dashed lines rTMS of right STG and dotted lines rTMS of the left STG. C) is the duration discrimination task E) is the frequency discrimination task. D–F) JND (black bars) and PSE (white bars) group data for temporal (D) and frequency (F) discrimination tasks as a function of TMS conditions. Asterisk indicates significant differences in Tukey’s t-tests (* p = 0.04).

To ensure anatomical accuracy, we used the Brainsight Frameless Stereotaxic system (Rogue Research, Montreal, Canada). Each subject was scanned to provide MRI T1 structural images. A Polaris (Northern Digital, Ontario, Canada) infra-red tracking device was used to measure the position of the subject’s head, and Brainsight software was used to co-register the subject’s head with the subject’s scan. The localization of left and right superior temporal gyri (STG) was based on the Talairach coordinates reported in Molholm et al. (2006). Coordinates for left STG were: x = −57, y = −14, z = 11; for right STG were x = 64, y = −26, z = 15. Statistical Parametric Mapping software (SPM2, Wellcome Department of Imaging Neuroscience, and University College London) was used to transform coordinates for left and right STG for each subject individually. This procedure involved normalizing each subject’s MRI scan against the standard MNI (Montreal Neurological Institute) template. The description of each resulting transformation was then used to convert the appropriate MNI coordinates to the untransformed (structural) space coordinates, yielding individual specific localization of the sites. These coordinates were then used to guide the frameless stereotaxy (see Figure 1 for rTMS sites of stimulation and Figure 2a for the timeline of the experimental paradigm).

### Data analysis

The primary dependent variables were: (1) percentage of accurate responses (number of hits/total number of observations per condition), (2) the Point of Subjective Equality (PSE) and (3) the Just Noticeable Difference (JND). The PSE is the point at which subjects, half of the time, judged the comparison duration to be longer than the standard. The JND was defined as the difference between estimated durations yielding 75% and 50% accuracy. For each individual participant, we estimated the point of subjective equality and the Just Noticeable Difference by fitting the subject’s performance in the longer/shorter (duration judgment) task, and in the higher/lower (frequency judgment) task with a psychometric function:
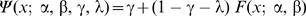
Where *x* is the comparison stimulus; α, β, γ, λ are the fitted model parameters which determine the shape of the psychometric function; and *F* is the Weibull function:
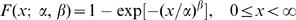



We used the *psignifit* toolbox (http://bootstrap-software.org/psignifit/) version 2.5.6 for Matlab (Mathworks, Inc) which implemented the maximum-likelihood method described by Wichmann and Hill [28] for curve fitting. In this way, PSE corresponds to the comparison stimulus for which the fitted model predicts 50% performance. JND is defined as the difference between the PSE and the comparison stimulus for which the fitted model predicts 75% performance. For each task we ran two separate one-way ANOVAs on PSE and JND values with Site (vertex, left STG, right STG) as the main factor.

We used the percentage of accurate responses (number of hits /total number of observations per condition) to run two separate ANOVAs, one for each task. Each Anova had site (rSTG, lSTG, and vertex) and Comparison Stimuli (±10, ±20 ±40 for the duration task and ±1, ±2 ±4 Hz for the frequency task) as main factors.

In all analyses post hoc comparisons were performed with the Tukey‘s test. Alpha level was set at 0.05.

The goodness of the fit was assessed by looking at the deviance (D) values resulting from the fit. Deviance is a ratio between the likelihood of a model with no residual error between empirical data and model predictions (*saturated* model) and the likelihood of the best-fitting model. Because deviance for binomial data is asymptotically distributed as χ^2^_k ,_ where k denotes the number of data points, each D value obtained from the fitting was compared with the appropriate χ^2^ distribution (χ^2^_6_ P <.025). For both tasks and all TMS conditions the model used to fit the data was not significantly different from the saturated model (duration experiment: D_mean_ = 3.99 D_stdev_ = 3.2; frequency experiment: D_mean_ = 4.61 D_stdev_ = 2.42 χ^2^_6_ P <.025).

## Results

The main finding of this experiment was a site specific and a task specific deficit following right STG TMS. When rTMS was applied over the right STG, temporal discrimination of auditory stimuli was significantly impaired (TMS Site effect on accuracy values F_2, 14_ = 4.98; P = .02). Compared to vertex and left STG stimulation, after TMS over right STG subjects were less accurate (Figure 2b) and required greater differences between the temporal standard and comparison stimuli to reach 75% accuracy (TMS Site effect on JND values F_2,21_ = 3.66; P = .04, Figure 2c and Figure 2d). No TMS effect was observed at any site for the frequency discrimination task (TMS Site effect on JND and accuracy values respectively F_2, 21_ = .37; P = .70; F_2, 14_ = .82; P = .46, Figure 2b,e–f.). TMS had no effects on the point of subjective equality at any site and in none of the tasks (duration task: TMS Site effect F_2,21_ = .32; P = .73, frequency tasks: TMS Site effect F_2,21_ = .55; P = .58, see Figure 2 d,f.)

## Discussion

In this study we showed that right superior temporal gyrus is important for timing of auditory stimuli. Magnetic stimulation affected the performance in two ways, lowering the overall accuracy and reducing the sensitivity compared to left superior temporal gyrus and vertex stimulation. Participants needed a bigger difference between standard and comparison durations in order to be able to discriminate them. The effect of magnetic stimulation on the just noticeable difference indicates that we interfered with the duration discrimination by increasing the uncertainty of the response rather then systematically biasing the perception of time towards the under or the overestimation. This effect is plausibly due to an increase of the neural noise in the stimulated areas induced by magnetic stimulation [29]. Moreover this result was not caused by an interference with low level auditory processing because magnetic stimulation did not affect participants ’ performance in the pitch discrimination task. Hemispheric asymmetries of the auditory cortex have been previously documented. A prevailing model is that temporal features, particularly relevant for speech analysis, are processed in the left hemisphere whereas spectral features, important for tonal analysis, are processed in the right [30]. This view has recently been challenged by an imaging study showing that both left and right auditory cortices are sensitive to the temporal structure of sounds, and that this sensitivity depends on the temporal window of the sound. Short range of duration (25–50 ms) are processed in the left and long range (>200 ms) in the right hemisphere [31]. It is possible, therefore, that the hemispheric asymmetry observed in our experiment is due to the range of duration used. The durations tested here were in the range of hundreds of milliseconds (from 560 to 640 ms), which, according to Boemio and collaborators, should be processed mainly in the right hemisphere.

Neuropsychological, neuroimaging and magnetic stimulation studies [6], [14], [15], [25], [32]–[34] have also reported a right hemisphere asymmetry for processing temporal information. These studies show a preferential involvement of parietal, dorsolateral, prefrontal and temporal cortices in the right rather than in the left hemisphere during both prospective and motor timing tasks [9]. Moreover, patients with right but not with left medial temporal lobe resection are impaired in the discrimination of auditory durations in the millisecond range [35] and in the retention of auditory but not visual rhythms [36]. Our result showing that an auditory area is necessary for timing of auditory events may thus be interpreted as demonstrating the existence of modality specific temporal processing. This idea is consistent with a theoretical position on timing that predicts modality specific timing in extrastriate visual areas [37], and is supported by psychophysics and neurophysiological studies in the visual modality [6], [38]–[40]. The contribution of modality specific and supramodal areas in temporal processing is a relevant but controversial issue in the understanding how and where time is represented in the brain. Assuming the existence of modality specific mechanisms implies that time is distributed in the brain [41], [42] and that many cortical areas are able to compute time depending, for example, on the task, the stimulus modality and whether the duration is in the range of milliseconds or seconds. However, it seems highly unlikely that this decentralization is absolute and that the modality specific mechanisms contain unique time generators. The interaction between sensory timing mechanisms and the cerebellar-basal ganglia networks [8], [43] as well as other cortical areas such as the parietal and prefrontal areas [22], [44], [45], remains to be resolved.
